# Supplementary material for: Liver transplantation for HCC with macrovascular invasion: A systematic review and meta-analysis of observational studies
Source: JHEP Rep. 2025 Aug 28;8(1):101566. doi: 10.1016/j.jhepr.2025.101566 (PMC12862345; doi:10.1016/j.jhepr.2025.101566)
Supplement: Multimedia component 1 [file mmc1.pdf]

Liver transplantation for HCC with macrovascular invasion: A systematic review and meta-analysis of observational studies☆

Farah Ladak, Christian Tibor Josef Magyar, Felipe D. Gaviria, Woo Jin Choi, Anudari Zorigtbaatar, Roxana Bucur, Nadia Rukavina, Arndt Vogel, Grainne Mary O'Kane, Zhihao Li, Marina Englesakis, Gonzalo Sapisochin

Table of content

Table S1 2

Table S1: Search Strategy

Search Topic: Hepatocellular Carcinoma and Liver Transplant and Macrovascular Invasion or Thrombus; limited to English language, human subjects. Conference and non-journal materials removed when possible.

Date Completed: Tuesday, January 24, 2023

MEDLINE

Ovid MEDLINE(R) 1946 to January 20, 2023

#	Searches	Results
1	Carcinoma, Hepatocellular/	101701
2	Adenoma, Liver Cell/	1015
3	Liver Neoplasms, Experimental/	15329
4	exp Liver Neoplasms/ and (adenoma* or carcinom* or hepatoma*).mp.	127106
5	(adult? adj1 liver? adj1 cancer*).mp.	10
6	(cancer* adj3 (liver* or hepat*)).mp,kf.	39958
7	(carcinom* adj3 (liver* or hepat*)).mp,kf.	127015
8	experimental hepatoma?.mp.	87
9	experimental hepat* neoplasm?.mp.	1
10	experimental liver neoplasm?.mp.	2
11	((hepatocellular or liver?) adj3 adenoma?).mp.	2533
12	(hepatocellular* adj2 carcinom*).mp.	122068
13	(liver? adj1 cell* adj1 adenoma*).mp.	1197
14	(liver? adj1 cell?? adj1 carcinom*).mp.	354
15	hepatocarcino*.mp,kw.	13026
16	hepato-carcino*.mp,kw.	128
17	HCC-MVI.mp.	13
18	morris?? hepatoma?.mp.	853
19	novikoff?? hepatoma?.mp.	483
20	or/1-19 [Hepatocellular Carcinoma & related terms]	174692
21	Liver Transplantation/	63393
22	exp Liver/tr [Use of "transplantation" floating subheading]	5
23	exp Liver Diseases/tr	6
24	exp Liver Diseases/ and exp Transplants/	1387
25	exp Liver/ and exp donor selection/	150
26	exp Liver/ and exp Graft Rejection/	1751
27	exp Liver/ and exp "Graft vs Host Disease"/	488
28	exp Liver/ and Tissue Donors/	1320
29	exp Liver/ and exp "Tissue and Organ Harvesting"/	577
30	exp Liver/ and exp "Tissue and Organ Procurement"/	578
31	exp Liver/ and exp Transplants/	745

32	exp Liver/ and exp Transplant Donor Site/	10
33	exp Liver/ and exp Unrelated Donors/	5
34	exp Liver/ and exp Vascular Grafting/	1638
35	exp Liver Diseases/ and exp Donor Selection/	558
36	exp Liver Diseases/ and exp Graft Rejection/	2918
37	exp Liver Diseases/ and exp "Graft vs Host Disease"/	694
38	exp Liver Diseases/ and exp "Tissue and Organ Harvesting"/	881
39	exp Liver Diseases/ and exp "Tissue and Organ Procurement"/	1750
40	exp Liver Diseases/ and exp Transplant Donor Site/	9
41	exp Liver Diseases/ and exp Unrelated Donors/	10
42	exp Liver Diseases/ and exp Vascular Grafting/	7960
43	Tissue Donors/ and (liver? or hepat*).mp.	7027
44	tr.fs. and (liver? or hepat*).mp. [Use of "transplantation" floating subheading]	6440
45	(deceased adj2 donor*).mp.	6925
46	(liver? adj3 transplant*).mp.	78878
47	(liver? adj3 allograft*).mp.	2898
48	(liver? adj3 autograft*).mp.	18
49	(liver? adj3 autotransplan*).mp.	132
50	(liver? adj3 auto-transplan*).mp.	6
51	(liver? adj3 graft*).mp.	6603
52	(living adj3 donor?).mp.	23409
53	(hepat* adj3 transplant*).mp.	10566
54	(hepat* adj3 allograft*).mp.	745
55	(hepat* adj3 autograft*).mp.	9
56	(hepat* adj3 autotransplan*).mp.	44
57	(hepat* adj3 auto-transplan*).mp.	4
58	(hepat* adj3 graft*).mp.	1317
59	(live adj3 donor?).mp.	2493
60	(liver? adj3 donor?).mp.	10886
61	(liver? adj3 donat*).mp.	863
62	(hepat* adj3 donor?).mp.	2389
63	(hepat* adj3 donat*).mp.	137
64	hemiliver?.mp.	165
65	hemihepatectom*.mp.	981
66	or/21-65 [Liver Transplantation & related terms]	117309
**67**	**20 and 66 [HCC + Liver Transplant]**	**12513**
68	exp Thrombosis/ and exp Portal Vein/	4755
69	exp Venous Thrombosis/	59323
70	thromb*.mp.	518841
71	HCC-MVI.mp.	13

72	PVTT.mp.	507
73	(arterial* adj3 inva*).mp.	1513
74	((artery or arteries) adj3 inva*).mp.	1999
75	(vasc* adj3 inva*).mp.	11133
76	(macrovasc* adj3 inva*).mp.	340
77	(venous adj3 inva*).mp.	2544
78	(macroscop* adj3 inva*).mp.	421
79	(blood adj2 clot*).mp.	10811
80	(major adj3 inva*).mp.	1383
81	or/68-80 [Thrombosis or Macrovascular Invasion]	549394
**82**	**67 and 81 [HCC + Liver Transplant + Thrombosis or Macrovascular Invasion]**	**1152**
	Limits applied:	
83	limit 82 to english language	1035
84	83 not (exp animals/ not (exp animals/ and exp humans/))	1027
85	limit 83 to humans	1027
86	84 or 85	1027
87	86 not (animal or animals or ape or apes or baboon or baboons or bat or bats or beagle or beagles or bird or birds or boar or boars or bonobo or bonobos or bovine or camel or camels or canine or canines or cat or cats or cattle or chick or chicks or chicken or chickens or chimpanzee or chimpanzees or dog or dogs or dromedary or dromedaries or duck or ducks or equine or equines or feline or felines or ferret or ferrets or frog or frogs or fowl or fowls or goat or goats or hamster or hamsters or hare or hares or hen or hens or horse or horses or lamb or lambs or livestock or macaque or macaques or mandrill or mandrills or mice or mink or minks or monkey or monkeys or mouse or murine or ovine or pig or pigs or piglet or piglets or poultry or porcine or orangutan or orangutans or rabbit or rabbits or rat or rats or rodent or rodents or sheep or spaniel or spaniels or swine or tamarin or tamarins or tiger or tigers or veterinary or veterinarian or veterinarians or waterfowl or waterfowls or weasel or weasels or veterinar* or fish or shellfish).ti,jw.	1023
88	86 not 87 [double check]	4
**89**	**87**<br>**[HCC + Liver Transplant + Thrombosis or Macrovascular Invasion; limited to English, humans]**	**1023**

Embase

Embase Classic+Embase 1947 to 2023 January 20

#	Searches	Results
1	exp liver cell carcinoma/ [Used for HCC in Embase]	198029
2	Carcinoma, Hepatocellular/ [MeSH]	112715
3	Adenoma, Liver Cell/	3301
4	exp liver cancer/ and (adenom* or carcinom* or hepatoma*).mp.	230402
5	exp Liver Neoplasms/ and (adenoma* or carcinom* or hepatoma*).mp.	240153
6	(adult? adj1 liver? adj1 cancer*).mp.	14

7	(cancer* adj3 (liver* or hepat*)).mp,kw.	95613
8	(carcinom* adj3 (liver* or hepat*)).mp,kw.	234254
9	experimental hepatoma?.mp.	116
10	experimental hepat* neoplasm?.mp.	1
11	experimental liver neoplasm?.mp.	328
12	((hepatocellular or liver?) adj3 adenoma*).mp.	5527
13	(hepatocellular* adj2 carcinom*).mp.	168492
14	hepatocarcino*.mp,kw.	19239
15	hepato-carcino*.mp,kw.	237
16	HCC-MVI.mp.	27
17	(liver? adj1 cell?? adj1 adenoma*).mp.	382
18	(liver? adj1 cell?? adj1 carcinom*).mp.	197983
19	morris?? hepatoma?.mp.	1197
20	novikoff?? hepatoma?.mp.	886
21	or/1-20 [Hepatocellular Carcinoma & related terms]	324113
22	Liver Transplantation/	116268
23	exp Liver Diseases/ and exp Transplants/	125050
24	exp Liver/ and exp donor selection/	208
25	exp Liver/ and exp Graft Rejection/	3759
26	exp Liver/ and exp "Graft vs Host Disease"/	2517
27	exp Liver/ and Tissue Donors/	7754
28	exp Liver/ and exp "Tissue and Organ Harvesting"/	14
29	exp Liver/ and exp "Tissue and Organ Procurement"/	46144
30	exp Liver/ and exp Transplants/	46144
31	exp Liver/ and exp Transplant Donor Site/	2188
32	exp Liver/ and exp Unrelated Donors/	96
33	exp Liver/ and exp Vascular Grafting/	792
34	exp Liver Diseases/ and exp Donor Selection/	938
35	exp Liver Diseases/ and exp Graft Rejection/	16283
36	exp Liver Diseases/ and exp "Graft vs Host Disease"/	6649
37	exp Liver Diseases/ and exp "Tissue and Organ Harvesting"/	15
38	exp Liver Diseases/ and exp "Tissue and Organ Procurement"/	125050
39	exp Liver Diseases/ and exp Transplant Donor Site/	5505
40	exp Liver Diseases/ and exp Unrelated Donors/	349
41	exp Liver Diseases/ and exp Vascular Grafting/	2126
42	Living Donors/ and (liver? or hepat*).mp.	14311
43	exp Tissue Donors/ and (liver? or hepat*).mp.	51644
44	(deceased adj2 donor*).mp.	20482
45	(liver? adj3 transplant*).mp.	152729

46	(liver? adj3 allograft*).mp.	4841
47	(liver? adj3 autograft*).mp.	28
48	(liver? adj3 autotransplan*).mp.	205
49	(liver? adj3 auto-transplan*).mp.	26
50	(liver? adj3 graft*).mp.	43775
51	(hepat* adj3 transplant*).mp.	18556
52	(hepat* adj3 allograft*).mp.	1015
53	(hepat* adj3 autograft*).mp.	18
54	(hepat* adj3 autotransplan*).mp.	74
55	(hepat* adj3 auto-transplan*).mp.	6
56	(hepat* adj3 graft*).mp.	2338
57	(live adj3 donor?).mp.	5927
58	(liver? adj3 donor?).mp.	22713
59	(liver? adj3 donat*).mp.	1901
60	(living adj3 donor?).mp.	43893
61	(hepat* adj3 donor?).mp.	4707
62	(hepat* adj3 donat*).mp.	261
63	hemiliver?.mp.	345
64	hemihepatectom*.mp.	2871
65	hemi*hepatectom*.mp.	2871
66	liver graft/ [Embase]	33330
67	exp deceased donor/	9186
68	exp liver/ and exp transplantation/ [Embase]	46144
69	exp liver/ and organ transplantation/	2545
70	exp liver disease/ and exp transplantation/	125050
71	exp liver disease/ and organ transplantation/	4318
72	exp liver resection/	17152
73	exp living donor/ and (liver? or hepat*).mp.	17510
74	or/22-73 [Liver Transplantation & related terms]	280285
**75**	**21 and 74 [HCC + Liver Transplant]**	**39680**
76	exp vein thrombosis/	162742
77	blood clot/	8255
78	deep vein thrombosis/	74163
79	lung embolism/	120708
80	thrombi.mp.	25421
81	thrombos*.mp.	449977
82	thrombot*.mp.	87129
83	((pulmonary or lung?) adj2 emboli*).mp.	133799

84	((artery or arteries) adj3 inva*).mp.	3474
85	(arterial* adj3 inva*).mp.	2916
86	(macro* adj3 inva*).mp.	4602
87	(major* adj3 inva*).mp.	3100
88	(vascular* adj3 inva*).mp.	20450
89	(portal vein? adj2 inva*).mp.	1544
90	PVTT.mp.	1091
91	HCC-MVI.mp.	27
92	(venous adj3 inva*).mp.	4314
93	(blood adj2 clot?).mp.	62378
94	exp thrombosis/	456994
95	or/76-94 [Thrombosis / Invasion]	717814
**96**	**75 and 95 [HCC + Liver Transplant + Vascular Invasion]**	**4353**
	Limits applied:	
97	limit 96 to english language	4184
98	97 not ((exp animals/ or exp animal experimentation/ or nonhuman/) not ((exp animals/ or exp animal experimentation/ or nonhuman/) and exp human/))	4149
99	limit 97 to human	3928
100	98 or 99	4149
101	100 [HCC + Liver Transplant + Vascular Invasion; limited to English, human]	4149
102	remove duplicates from 101 [removal of internal duplicate citations]	4057
103	limit 102 to (conference abstracts or "preprints (unpublished, non-peer reviewed)" or (books or chapter or conference abstract or conference paper or "conference review" or "preprint (unpublished, non-peer reviewed)") or (book or book series or conference proceeding or "preprint archive (unpublished, non-peer reviewed)" or trade journal))	1629
104	102 not 103 [removal of conference and non-journal material]	2428
**105**	**104 [HCC + Liver Transplant + Vascular Invasion; limited to English, human, journal articles]**	**2428**
