# Supplementary material for: Liver transplantation for HCC with macrovascular invasion: A systematic review and meta-analysis of observational studies
Source: JHEP Rep. 2025 Aug 28;8(1):101566. doi: 10.1016/j.jhepr.2025.101566 (PMC12862345; doi:10.1016/j.jhepr.2025.101566)
Supplement: Multimedia component 4 [file mmc4.pdf]

Liver transplantation for HCC with macrovascular invasion: A systematic review and meta-analysis of observational studies☆

Farah Ladak[1,†], **Christian Tibor Josef Magyar**[1,2,†], Felipe D. Gaviria[1], Woo Jin Choi[1], Anudari Zorigtbaatar[1], Roxana Bucur[1], Nadia Rukavina[1], Arndt Vogel[3,4,5], Grainne Mary O'Kane[5,6,7], Zhihao Li[1], Marina Englesakis[1], Gonzalo Sapisochin[1,*]

JHEP Reports **2026**. vol. 8 | 1–11

Check for updates

Background & Aims: Traditional guidelines discourage liver transplantation (LT) in patients with hepatocellular carcinoma (HCC) with macrovascular invasion (MVI). However, emerging evidence suggests that long-term survival is possible when LT is preceded by downstaging therapies. Thus, a pooled analysis of time-dependent risk factor effect size on overall survival (OS) is warranted to determine the effect size of MVI, estimate risk mitigation through downstaging, and design future trials.

Methods: MEDLINE, EMBASE, Cochrane Database of Systematic Reviews, and the Cochrane Register were systematically searched from their inception to 24 January 2023. Studies with comparative oncological outcome data between patients with HCC and MVI (MVI group) and HCC patients without MVI (non-MVI group) were included. Frequentist pairwise meta-analysis (random-effects model) was performed for primary outcomes of OS and recurrence-free survival (RFS) at 5 years. Only studies with adjusted effect estimates were considered for analysis.

Results: In total, 10 studies were included in this systematic review and meta-analysis, contributing 15,899 patients. Seven studies included deceased donor LT, and four studies included living donor LT. In the quantitative analysis of studies reporting adjusted effect estimates, presence of MVI was associated with lower 5-year OS (hazard ratio (HR) 2.03; 95% CI 1.60–2.57; p <0.001; I^2 = 55%; p = 0.05) and 5-year RFS (HR 2.55; 95% CI 1.69–3.85; p <0.001; I^2 = 87%; p <0.01). When downstaging was uniformly applied, no statistically significant difference for 5-year OS was observed between the MVI group and non-MVI group (HR 1.55; 95% CI 0.88–2.73; p =0.129; I^2 = 46%; p = 0.17).

Conclusions: Effective downstaging in carefully selected patients with HCC with MVI could achieve survival outcomes approaching those of patients without MVI. Further studies are essential to validate these findings and to clarify which downstaging approaches and tumor characteristics are most likely to confer a transplant benefit.

© 2025 The Authors. Published by Elsevier B.V. on behalf of European Association for the Study of the Liver (EASL). This is an open access article under the CC BY license (http://creativecommons.org/licenses/by/4.0/).

Introduction

Macrovascular invasion (MVI) is detected in 10–35% of patients with hepatocellular carcinoma (HCC) at diagnosis and heralds a dismal prognosis.[1] Median overall survival (mOS) is 2–5 months with best supportive care, owing largely to rapid intrahepatic progression and systemic dissemination with portal and hepatic vein invasion.[1,2] Moreover, MVI heightens the risk of adjacent tumor thrombus (*e.g.* portal vein tumor thrombus [PVTT]) formation, exacerbating liver dysfunction and limiting therapeutic avenues.[3]

The Barcelona Clinic Liver Cancer (BCLC) Staging System classifies portal vein and hepatic vein tumor thrombus or invasion as advanced disease (BCLC C), for which systemic therapy is the recommended treatment.[4] Until 2017, treatment options were limited to tyrosine kinase inhibitors, offering modest benefits at best. The advent of immune checkpoint inhibitors and anti-angiogenic agents has since transformed the therapeutic landscape, creating new possibilities for downstaging previously unresectable tumors. First-line therapies for patients with advanced disease and preserved liver function have shown remarkable survival benefits. For example, in the IMBrave150 trial, atezolizumab plus bevacizumab achieved an mOS of 19.2 months (95% CI 17.0–23.7), while, in the HIMALAYA trial, tremelimumab plus durvalumab demonstrated an mOS of 16.4 months (95% CI 14.2–19.6).[5,6] Subgroup analysis from the IMBrave150 trial further highlighted survival improvements, with atezolizumab plus bevacizumab achieving an mOS of 7.6 months (95% CI

☆ Given their role as Co-Editor, AV had no involvement in the peer-review of this article and had no access to information regarding its peer-review. Full responsibility for the editorial process for this article was delegated to the Guest Editor, Tim Meyer.

* Corresponding author. Address: HBP & Multi Organ Transplant Program, Division of General Surgery, University Health Network, 585 University Avenue, 9-MaRS-9047B, Toronto, ON, M5G 2N2, Canada. Tel.: +1 416 340 4800 ext. 5169; fax: +1 416 340 3237.
E-mail address: gonzalo.sapisochin@uhn.ca (G. Sapisochin).
† Shared co-first authorship.

https://doi.org/10.1016/j.jhepr.2025.101566

6.0–13.9) in patients with main portal vein trunk invasion (Vp4).[7] Moreover, emerging therapies investigated in the CARES-310 and Checkmate 9DW trials[5,6,8–11] have shown encouraging results, with radiological complete response rates ranging from 3.1% to 7.7%, paving the way for additional treatment options in this challenging patient population.

Surgery, including liver resection and liver transplantation (LT), are not generally recommended in patients with MVI (especially in Western countries) because of historically higher rates of recurrence and reduced overall survivial (OS) compared with those without MVI.[3,12–14] However, emerging evidence suggests that downstaging is possible in selected patients receiving locoregional and systemic therapy. LT following downstaging is both safe and feasible, with promising outcomes.[15] Liu *et al.* provide a descriptive analysis of the evidence to date, evaluating LT for patients with locally advanced HCC. The pooled 5-year OS was 49% (95% CI 39–58; I^2 = 78.4%) in all patients and 63% (95% CI 53–73%; I^2 = 0%) in the subgroup of patients with 'successful downstaging'.[16] Although reporting proportions has value, they cannot be used to determine the effect size related to specific factors or time-to-event outcomes. To date, no meta-analysis has provided the pooled adjusted hazard ratio (aHR) for the impact of MVI on outcomes after LT. This lack of data creates uncertainty regarding the effect size of MVI on post-transplant outcomes and the potential for risk mitigation through downstaging, which is essential for designing and powering future trials.

In this study, we assessed differences in OS and recurrence-free survival (RFS) in patients with MVI and HCC undergoing LT following downstaging treatment compared with those without MVI. Where possible, we characterized downstaging treatment and delineated differences in study protocols, with a view to standardizing outcome reporting and reducing study heterogeneity.

Materials and methods

This systematic review adheres to the Preferred Reporting Items for Systematic Reviews and Meta-Analyses (PRISMA) statement guidelines.[17]

Information sources and search strategy

The search strategies were developed by a health science librarian (ME) with experience in systematic reviews and meta-analyses. The following databases were searched from inception via the Ovid platform: MEDLINE, MEDLINE ePubs and In-Process Citations (daily), Embase Classic+Embase, Cochrane Database of Systematic Reviews, and the Cochrane Central Register of Controlled Trials. All databases were searched on the same day, 24 January 2023.

The search process followed the Cochrane Handbook[18] and the Cochrane Methodological Expectations of Cochrane Intervention Reviews (MECIR)[19] for conducting the search, PRISMA 2020 for reporting, and PRISMA-S[20] extension for searches. The PRESS guideline for peer-reviewing the search strategies[21] was used, drawing upon the PRESS 2015 Guideline Evidence-Based Checklist to avoid potential search errors. Preliminary searches were conducted, and full-text literature was mined for potential keywords and appropriate controlled vocabulary terms (*e.g.* Medical Subject Headings [MeSH] for MEDLINE, and EMTREE descriptors for Embase). The Yale MeSH Analyser was used to facilitate the MeSH and text word analysis. The search strategy concept blocks were built on the topics of: '(Hepatocellular Carcinoma) AND (Macrovascular Invasion OR Thrombus) AND (Liver Transplant)' using both controlled vocabularies and text word searching for each component.[22] Searches were limited to English language, and human subjects. Where possible, conference and non-journal materials were removed from results at source. Following full-text assessment, hand-search of other potential sources, including Google Scholar, did not identify additional papers. The Ovid MEDLINE search strategy is provided in Table S1.

Eligibility criteria and study selection

All studies that provided comparative outcome data on patients with HCC and MVI treated with downstaging therapy followed by LT and patients with HCC without MVI treated with LT, were included. MVI was defined as invasion of the branches of the right, left, or main portal vein or as invasion of the right, middle, or left hepatic vein. We excluded: (1) patients with concurrent cholangiocarcinoma; (2) single-arm studies with downstaging therapy for MVI alone; (3) case series with fewer than eight cases; (4) animal studies; and (5) review articles. All abstracts were independently screened for relevance by two authors (FL and FDG and/or CTJM) using covidence.org. Articles selected for full-text review were independently appraised (FL and CTJM), using predefined eligibility criteria. Discordant assessments at each step were reconciled with contribution from a third senior reviewer (GS).

Data collection and endpoints

Baseline characteristic and outcome data were extracted using a piloted, standardized template designed by the authors. Two independent authors collected the data. Variables of interest were identified before the review search, including demographic (geographic region, age, and sex), clinical/laboratory (alpha-fetoprotein [AFP], model of end-stage liver disease [MELD], and etiology of liver disease), and pathological variables (tumor number/size, grade, and presence of micro- or macrovascular invasion). Outcomes were collected separately. The primary and secondary outcomes were OS and RFS, respectively. Effect estimates, including 95% CI and the number of patients in each treatment arm, were collected.

Risk of bias within individual studies

Individual study bias was assessed using the Newcastle-Ottawa Scale (NOS),[23] which scores each article based on the following: (1) representativeness of the exposed cohort; (2) ascertainment of exposure; (3) demonstration that the outcome of interest was not present at the start of the study; (4) comparability of cohorts on the basis of the design or analyses; (5) assessment of outcome; (6) was follow-up long enough for outcomes to occur; and (7) adequacy of follow-up of cohorts. A good-quality study was defined by a total score ≥7. Fair and poor-quality studies were defined by a score of 4–6 and ≤3, respectively.

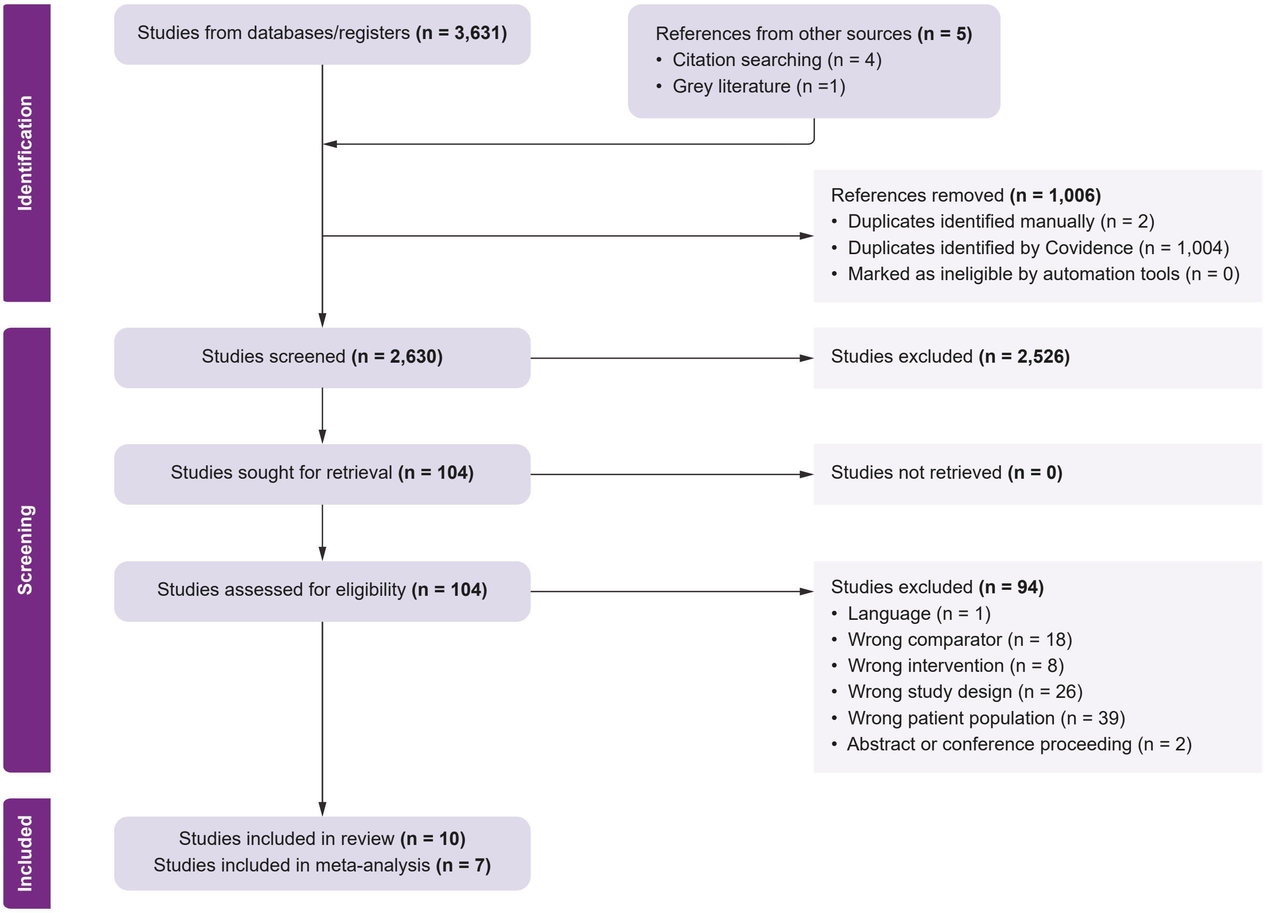

Fig. 1. PRISMA flowchart. PRISMA, Preferred Reporting Items for Systematic Reviews and Meta-Analyses.

Summary measures and methods of analysis

Our meta-analysis was limited to studies where an aHR was available. A random-effects model was used in anticipation of heterogeneity across studies and the statistical heterogeneity was assessed using the I^2 statistical estimate. I^2 was categorized as: low, <30%; moderate, 30–50%; substantial, 50–75%; and considerable, >75%.[24] Subgroup analysis was performed to discern the impact of downstaging before LT in patients with HCC with MVI, using only those studies where successful and uniform downstaging were reported. If studies were unclear around the use of downstaging therapy, they were grouped for a separate subgroup analysis. All analyses were conducted in R version 4.3.2 using 'meta' package (R Foundation for Statistical Computing, Vienna, Austria).[25]

Results

Between inception and 24 January 2023, our search yielded a total of 3,631 citations (Fig. 1). After removing duplicates, 2,630 studies remained for abstract review, of which 104 studies were deemed eligible for full-text review (Fig. 1).

Qualitative results

Ten articlesmet our inclusion criteria, contributing a combined study population of 15,899 patients (Table 1).[15,26–34] Included studies originated from Austria, China, Europe, India, Italy, Japan, Korea, and the USA (Table 1), with accrual time periods ranging from 1988 to 2022. Systemic therapy was not reported in any patients. The mOS was reported in two studies as 4.1 and 14.4 years and 5-year OS was reported in nine studies, ranging from 34.8% to 79.5% (Table 2). The NOS for assessment of the risk of bias total score ranged from 3 to 8 points (Table 3). Two studies were considered poor quality (≤3 total score) (D'Amico *et al.* 2009 and Todo *et al.* 2004),[26,34] six studies were fair quality,[15,27–29,31,32] (4–6 total score) and two studies were good quality (≥7 total score) (Yu *et al.* 2022 and Soin *et al.* 2020).[30,33]

Two studies (Finkenstedt *et al.* and Yu *et al.*) reported survival rates for patients receiving downstaging therapy: Finkenstedt *et al.* performed a subgroup analysis of 23 patients with BCLC stage C.[32,33] All patients had MVI or extrahepatic tumors and three (13%) had poorly differentiated tumors on explant histopathology. All but one patient (22/23) received pre-LT treatment (resection, radiofrequency ablation, and/or transarterial chemoembolization [TACE]) with a pathological complete response (pCR) observed in 11% (two of 19), partial response in 68% (13/19), and progression in 21% (four of 19). Median OS was determined to be 4.1 years (95% CI 2.9–5.2), while median RFS was 3.1 years (95% CI 2.0–4.1). On multivariate assessment, MVI/extrahepatic tumor was associated with a decrease in OS after LT (aHR 2.40; 95% CI 1.04–5.57; p = 0.040).

Table 1. Systematic review study-level data: characteristics and patient information.

First author (year, country)	Study period, design, setting	Inclusion/exclusion criteria	Arm	No. patients	Downstaging modality	Age, y/male (%)	MELD	HBV/HCV/ALD/NASH, MASLD	Tumor size (median, cm)/number/AFP (ng/ml)	MC	Response rate on final pathology n (%)	Poor tumor differentiation (%)/Microvascular invasion (%)	Macrovascular invasion (%)	Median follow up time (months)
Finkenstedt (2016, Austria)	2002-2013, retrospective, Single center	I: LT for HCC E: -	MVI (BCLC C)	23	94% received pre-transplant tx. TACE:121 (70%), RFA: 60 (35%), LR:9 (5%). TACE/RFA used for BCLC C	58.9 (47.2–68.7)/78%	10 (7)	2 (6%)/10 (29%)/11 (32%)/7 (21%)	9.0 (2.1–22.4)/2.7 (1–9)/6.4 (33.3)		pCR:2 (9%) partial:13 (57%) progression: 4 (17%)	3 (13%)/3 (13%)	23 (100%)	-
			BCLC 0/A	103		60.8 (34.0–75.2)/90%	11 (7)	7 (7%)/35 (34%)/42 (41%)/15 (14%)	3.0 (1.1–6.2)/1.3 (1–3)/5.0 (12.0)		pCR: 47 (46%)	3 (3%)/4 (4%)		
			BCLC B	48		61.8 (41.4–70.8)/90%	11 (6)	6 (13%)/11 (23%)/16 (33%)/11 (23%)	8.1 (4.8–2.02)/2.8 (1–9)/5.1 (10.6)	-		2 (4%)/4 (9%)	-	-
D'Amico (2009, USA, Italy)	1988-2007, Retrospective, Multicentric	I: LT for HCC E: Incomplete histopathology	Within Up-to 7 Criteria	355	Liberal pre-transplant protocol	56 (27–76)/270 (76.1%)		56 (17%)/236 (66%)/33 (9%)/-	2.5 (0.2–6.0)/2 (1–5)/21 (1–61,720)	-		26 (7%)/89 (25%)	0 (0%)	-
			Beyond Up-to-7 Criteria	124	Liberal pre-transplant protocol	58 (19–75)/104 (83.9%)		19 (15%)/75 (60%)/19 (15%)/-	5.0 (1.0–15.0)/3 (1–15)/94 (4–39,083)	-		22 (18%)/49 (40%)	36 (29%)	-
Pommergaard (2018, Europe)	1990-2016, Prospective, Multicenter	I: LT for HCC E: Without cirrhosis Without data on explant	No vascular invasion	5,885	-	56.8 (8.6)/5,005 (85.0%)	-	-/-/-/-	-/-/-	-		-/-	-	23 (0-289)
			Macrovascular invasion	231	-	56.5 (9.3)/196 (84.8%)	-	-/-/-/-	-/-/-	-		-/-	-	
Kim (2023, South Korea)	2009-2022, Retrospective, Single center	I: liver-directed combined radiotherapy and LT E: Within Milan	-	55	Liver directed combined radiotherapy	-/-	-	-/-/-/-	-/-/-	0%	33/39 (85%)	-/-	-	48.6 (6.9 – 151.7)
Pommergaard (2018, Europe)	1990-2016, Retrospective, Registry	I: LT for HCC E: Missing data	LRT	3572	TACE 59.1% RFA 18% Resection 4.7% RFA+TACE 7.8%	58 (0–77)/3,030 (84.8%)	10.1 (6.4–42.8)	-/-/-/-	-/-/-	2,429 (68%)		-/536 (18.6%)	83 (2.9%)	-
			No LRT	1406	-	55 (0–78)/1,151 (81.9%)	12.1 (6.4–49.6)	-/-/-/-	-/-/-	875 (62%)		-/254 (20.4%)	59 (4.7%)	-

(*continued on next page*)

Table 1. (*continued*)

First author (year, country)	Study period, design, setting	Inclusion/ exclusion criteria	Arm	No. patients	Downstaging modality	Age, y/ male (%)	MELD	HBV/HCV/ ALD/ NASH, MASLD	Tumor size (median, cm)/num- ber/AFP (ng/ml)	MC	Response rate on final pathology n (%)	Poor tumor differ- entiation (%)/Microvascular invasion (%)	Macrovascular invasion (%)	Median follow up time (months)
Soin (2020, India)	2006-2017, Prospective, Single center	I: LDLT data- base E: Non-HCC	HCC-cirr, PVTT, LDLT post- downstaging	25*	SBRT w/ cyberknife + ablation w/ TARE/TACE/ RFA/MWA	51 ± 8/23 (92%)	10 (6–21) 10	11 (44%)/9 (32%)/2 (8%)/0 (0)	6.3 ± 5.2/2.9 ± 1.6/55 (2–7,320)	4 (16%)	PVTT necro- sis >50%: 21/25 (82%); pCR 3/25 (12%)	-/21 (84%)	PVTT Vp1:Vp2: Vp3:Vp4 1 (4%):12 (48%): 11 (44%):1 (4%	33 (2-86)
			HCC-cirr, PVTT, LDLT without downstaging	21	-	58 ± 7/19 (90%)	11 (6–25)	3 (14%)/10 (48%)/3 (14%)/1 (5%)	5.1 ± 3.2)/ 2.9 ± 1.9/ 271 (4–17,104)	8 (38%)		-/17 (81%)	PVTT Vp1:Vp2: Vp3:Vp4 5 (23.8%):13 (61.9%): 3 (14.3):0 (0)	-
			HCC-cirr, no PVTT, LDLT	405	-	55 ± 8/322 (80%)	19 (6–38)	104 (26%)/ 183 (45%)/ 40 (10%)/35 (9%)	4.3 ± 3.2/2.0 ± 1.3/23.7 (1–17,500)	197 (49%)		-/164/326 (50%)	-	-
Sha (2022, China)	2015-2019, Retrospective, Single center	I: DDLT for HCC E: Other than HCC, Periop- erative death, loss to FU within 90 days of transplant Incomplete medical records	Control	156	Pretransplant treatment:37 (23.7%)	53 (29-74)/ 133 (85.3)		146 (93.6%)/-/-/-	3.5 (0.3- 24)/-/20.2 (1.3-60,500)	-		36 (23.1)/-	-	-
			PVTT	46	Pretransplant treatment:11 (23.9%)	51 (34-66)/ 40 (87.0)		41 (89.1%)/-/-/-	7.5 (1-15)/-/ 105.6 (0.7- 60,500)	-		23 (50.0)/-	-	-
Tabrizian (2022, USA)	2001-2015, Prospective, Multicenter	I: LT for HCC E: -	Within Milan	2122	Pretransplant treatment: 1,780 (83.8%)	60 (55- 64.9)/1,604 (75.6)	12 (9-17)	297 (14%)/ 1299 (61.2%)/177 (8.3%)/107 (5%)	1.8 (0-3)/1 (1-2)/8.4 (4.7-27.6)	100%		216 (10.2)/382 (18)	49 (2.3)	55.3 months (IQR, 26.4,93.5)
			Downstaged to within MC	341	Pretransplant treatment: 341 (100%)	59.4 (54.8- 64.2)/279 (81.8)	11 (8-15)	64 (18.8%)/ 182 (53.4%)/28 (8.2%)/21 (6.2%)	4.5 (3.4-5.7)/ 2 (1-2)/10 (5- 42.6)			39 (11.4)/69 (20.2	14 (4.1)	
Todo (2004, Japan)	1989-2003, retrospective, national regis- try, 29 centers	I: LDLT for HCC E: No malig- nancy on pa- thology, ne- crosis after therapy, lost to FU, non-adult	overall cohort, single arm	316	Pretransplant treatment: 232 (73.4%)	54 years (25–70)/246 (77.8%)	≤10: 116 (36.7%)	99 (31.3%)/ 182 (57.6%)/28 (8.9%).	≤2 cm: 116 (36.7%) 2-5: 161 (50.9%) >5 cm: 29 (9.2%)/Soli- tary 75 (23.7%)/≤20: 125 (39.6%)	-		51 (16.1%)/-	Vp1: 46 (14.6%) Vp2: 13 (4.1%) Vp3: 8 (2.5%)	16 months (2.5-% 72.0)

(*continued on next page*)

Table 1. (*continued*)

First author (year, country)	Study period, design, setting	Inclusion/ exclusion criteria	Arm	No. patients	Downstaging modality	Age, y/ male (%)	MELD	HBV/HCV/ ALD/ NASH, MASLD	Tumor size (median, cm)/number/AFP (ng/ml)	MC	Response rate on final pathology n (%)	Poor tumor differentiation (%)/Microvascular invasion (%)	Macrovascular invasion (%)	Median follow up time (months)
Yu (2022)	2015-2018, Retrospective, Multicenter	I: LT for HCC E: <18 years, lost to FU within 90 days of LT, incomplete records	without PVTT; within Milan	489	Pretransplant locoregional 181 (37.0)	52.4 ± 9.1/ 441 (90.2)	16.0 (9.0-31.0)	437 (89.4%)/-/-/-	2.5 (1.7-3.2)/ Solitary: 337 (68.9%)/18.2 (4.0-143.4)	-		48 (9.8)/-	-	25.3 months
			PVTT type 1-2	176	Pretransplant locoregional (RFA/TAACE/ PEI)69 (39.2)	50.8 ± 10.2/162 (92.0)	13.0 (9.0-28.0)	163 (92.6 %)/-/-/-	5.0 (3.5-8.0)/ Solitary: 107 (60.8%)/ 178.4 (10.7-1,732.2)	-		45 (25.6)/-	-	

*Soin *et al*. Downstaging was applied to 43 patients with HCC + PVTT, of whom 27 had a radiologically successful response, defined as absence of contrast enhancement in the tumor thrombus on CT or loss of FDG avidity on PET. Of the 27 patients, 25 underwent LDLT. The remaining two patients did not proceed for psychological reasons and inability to locate a suitable donor. In the 16 patients who were not successfully downstaged, nine showed continued tumor activity, and seven progressed to metastatic disease. AFP, alpha-fetoprotein; ALD, alcohol-related liver disease; BCLC, Barcelona Clinic Liver Cancer; CT, computed tomography; DDLT, deceased donor liver transplantation; FU, follow-up; HCC, hepatocellular carcinoma; LDLT, living donor liver transplantation; LRT, locoregional treatment; LT, liver transplantation; MC, Milan criteria; MELD, model of end-stage liver disease; MVI, macrovascular invasion; pCR, pathologic complete response; PEI, percutaneous ethanol injection; PET, positron emission tomography; PVTT, portal vein tumor thrombus; pCR, pathological complete response; RFA, radiofrequency ablation; SBRT, stereotactic radiotherapy; TACE, transarterial chemoembolization; TARE, transarterial radioembolization.

Yu *et al.* provide crucial insights into the prognostic implications of PVTT level on OS.[33] Between 2015 and 2018, 176 patients with locally advanced HCC underwent LT. Patients were stratified based on PVTT involvement: segmental (Cheng's type 1 PVTT; n = 83) *vs.* lobar (Cheng's type 2 PVTT; n = 93).[35] Preoperative LRT was given to 39% patients. Five-year OS was higher in patients with segmental PVTT compared with those with lobar involvement (78.3% *vs.* 51.65%). However, on multivariable analysis, the presence of PVTT, irrespective of type, was not significantly associated with OS (aHR 1.283; 95% CI 0.922–1.78; p = 0.139).

A post hoc analysis of 5-year OS between Cheng's type 1 PVTT (78.3%, 65/83) and Cheng's type 2 PVTT (51.6%, 48/93) demonstrated a trend favoring improved survival for type 1 PVTT; however, this difference did not reach statistical significance (odds ratio (OR) 1.52; 95% CI 0.94–2.44; p = 0.086). Notably, the authors reported that adjuvant sorafenib therapy was recommended at some centers; however, data regarding its use and duration were not provided and, consequently, this variable was excluded from the multivariable analysis.

AFP levels were significantly associated with OS, with patients having AFP >100 ng/ml experiencing worse outcomes compared with those with AFP <100 ng/ml (aHR 1.843; 95% CI 1.374–2.473; p <0.001).

Quantitative results: meta-analysis

The adjusted effect (reported or estimated HRs) comparing patient cohorts were available in seven publications, contributing 10,264 patients.

Overall survival

Six studies compared 5-year OS between the MVI and non-MVI group.[15,27,31–34] Post-LT OS was significantly lower in patients with MVI compared with non-MVI (HR 2.03; 95% CI 1.60–2.57; p <0.001) (Fig. 2A). Of note, statistical heterogeneity was substantial and significant (I^2 = 55%; p = 0.05). Tumor characteristics are summarized in Table 1.

Downstaging and overall survival

Two studies (Finkenstedt *et al.* and Yu *et al.*) reported survival rates for patients receiving downstaging therapy.[32,33] Subgroup analysis found no significant difference in 5-year OS between patients with MVI who had undergone preoperative downstaging compared with patients without MVI (HR 1.55; 95% CI 0.88–2.73; p = 0.129; Fig. 2B). The statistical heterogeneity between these two studies was moderate and not significant (I^2 = 46%; p = 0.17).

No downstaging and overall survival

In the complimentary subgroup, where explicit reference to downstaging was not provided (four studies), 5-year OS in the MVI group was significantly decreased (HR 2.26; 95% CI 2.09–2.43; p <0.001; Fig. 2C).[15,27,31,34] Statistical heterogeneity was low and not significant (I^2 = 0%; p = 0.95).

Recurrence-free survival

Six studies provided 5-year RFS data.[15,26,27,31–33] RFS was significantly shorter in patients with MVI relative to those

Table 2. Systematic review study-level data: survival data.

First author	Year	Journal	Country	Study period	Arm	No. of patients	Median FU (IQR)	Median OS (IQR)	1-year OS	3-year OS	5-year OS (IQR)
Finkenstedt[32]	2016	Liver International	Austria	2002–2013	MVI (BCLC C)	23	–	4.1 years (2.9–5.2)	–	–	56%
					BCLC 0/A	103	–	9.3 years (8.0–10.6)	–	–	77%
					BCLC B	48	–	8.7 years (7.8–9.6)	–	–	79%
D'Amico[26]	2009	Liver Transplantation	USA, Italy	1988–2007	Within up-to-7 MC	355	32.2 months	–	82%	67%	61%
					Beyond up-to-7 MC	124		–			
Pommergaard[27]	2018	HPB	Europe	1990–2016	No vascular invasion	5,885	23 months (0–289)	–	–	–	70.7% (71.9–69.5)
					MVI	231	–	–	–	–	39.6% (32.5–46.7)
Kim[28]	2023	International Journal of Radiation Oncology Biology Physics	Korea	2009–2022	–	55	48.6 months (6.9 –151.7)	–	–	–	38.1%
Pommergaard[29]	2018	Transplant International	Europe	1990–2016	LRT	3572	33–44 months	–	–	–	51.3–80.9%
					No LRT	1406	26 months	–	–	–	65.8%
Soin[30]	2020	Transplantation	India	2006–2017	HCC-cirr, PVTT, LDLT post downstaging	25	33 months	NE	82%	57%	57%
					HCC-cirr, PVTT, LDLT without downstaging	21	–	–	80%	59%	48%
					HCC-cirr, no PVTT, LDLT	405	–	NE	94%	80%	65%
Sha[31]	2022	Frontiers in Oncology	China	2015–2019	Control	156	–	–	93.6%	82.1%	79.5%
					PVTT	46	–	–	80.4%	37%	34.8%
Tabrizian[15]	2022	JAMA Surgery	USA	2001–2015	Within MC	2,122	55.3 months	172.8 m	89.4%	-	73.6%
					Downstaged to within MC	341	(26.4–93.5)	126.0 m	90.1%	-	67.9%
Todo[34]	2004	Annals of Surgery	Japan	1989–2003	Within MC	137	16 months (2.5–72.0)	–	82%	79.4%	–
					Beyond MC	172		–	74.5%	60.0%	–
Yu[33]	2022	EJSO	China	2015–2018	Without PVTT; within MC	489	25.3 months	–	93.3%	85.3%	79.1%
					PVTT type 1	83	–	–	88.0%	78.3%	78.3%
					PVTT type 2	93	–	–	77.4%	51.6%	51.6%

BCLC, Barcelona Clinic Liver Cancer; cirr, cirrhosis; FU, follow-up; HCC, hepatocellular carcinoma; LDLT, living donor liver transplantation; LT, liver transplantation; MC, Milan Criteria; MVI, macrovascular invasion; PVTT, portal vein tumor thrombus.

Table 3. Newcastle-Ottawa scale for assessment of risk of bias of included studies.

	Selection			Comparison	Outcome			
Author (year of publication)	Representativeness of exposed cohort	Ascertainment of exposure	Demonstration that outcome of interest was not present at start of study	Comparability of cohorts on basis of design or analyses	Assessment of outcome	Was follow-up long enough for outcomes to occur	Adequacy of follow-up for outcomes to occur	Total
Finkenstedt *et al.* (2016)[32]	*	–	*	*	*	–	*	6
D'Amico *et al.* (2009)[26]	*	–	*	–	*	–	–	3
Pommergard *et al.* (2018)[27]	*	–	*	*		*	*	5
Kim *et al.* (2023)[28]	*	*	*	–	*	–	–	4
Pommergaard *et al.* (2018)[29]	*	*	*	–	–	*	–	4
Soin *et al.* (2020)[30]	*	*	*	**	*	*	*	8
Sha *et al.* (2022)[31]	*	–	*	–	*	*	–	4
Tabrizian *et al.* (2022)[15]	*	–	*	–	*	*	*	5
Todo *et al.* (2004)[34]	*	–	*	–	*	–	–	3
Yu *et al.* (2022)[33]	*	–	*	*	*	*	*	6

without (HR 2.55; 95% CI 1.69–3.85; p <0.001; Fig. 3). Statistical heterogeneity between studies was considerable and significant (I^2 = 87%; p <0.01).

Only one study provided explicit reference to downstaging therapy in the MVI group. Finkenstedt *et al.* reported the RFS between groups to be significantly different (aHR 4.86; 95% CI 2.17–19.82; p = 0.001).[32]

Discussion

To the best of our knowledge, this meta-analysis provides the first systematic comparison of oncological outcomes between patients undergoing LT with locally advanced HCC defined by the presence or absence of MVI. Our findings demonstrate that patients with MVI experience lower OS and RFS (HR 2.03; 95% CI 1.60–2.57) relative to those without. However, when restricted to patients with MVI who were successfully downstaged, no statistical significant difference in OS was observed between cohorts (HR 1.55; 95% CI 0.88–2.73). However, there were insufficient data to determine whether the same held true for RFS.

Our study highlights that downstaging therapy can effectively improve post-transplant OS and RFS, potentially mitigating the poor prognostic impact of MVI in a selected group of patients. These findings expand upon the results of Liu *et al.*, who recently reported on the transplant benefit in locally advanced HCC following downstaging therapy in a series of single-arm studies.[16] Downstaging therapy might halt tumor progression via immune-mediated reactions and/or reflect favorable tumor biology. Although protocols for downstaging remain undefined, there have been promising results with stereotactic radiotherapy with or without transarterial chemoembolization, resulting in radiologically complete resolution of PVTT in select patients.[30,36] As we look toward future studies, several key questions must be addressed: (1) Which downstaging protocol is most effective for treating PVTT? (2) How should response to therapy be defined? (3) What constitutes successful downstaging? (4) Does achieving complete radiological resolution offer a distinct transplant benefit, or would a partial response or the absence of disease progression suffice to justify transplantation? And (5) most importantly, what is the minimum acceptable 5-year OS rate to warrant organ allocation, acknowledging regional differences in organ scarcity?[37]

Although our meta-analysis showed comparable survival in patients with HCC with and without MVI following downstaging, there is limited but important evidence to suggest a potential difference in outcomes based on the location/extent of vascular involvement. Only one of the included studies addressed the difference in outcomes for lobar *vs.* segmental PVTT involvement. Patients with lobar PVTT exhibit significantly higher rates of recurrence and poorer OS compared with those with segmental involvement alone. This discrepancy becomes more pronounced when juxtaposed with patients with microvascular invasion or those lacking vascular invasion entirely. Notably, none of the included studies addressed hepatic vein involvement, which, according to natural history studies, might share a similar tumor biology to lobar PVTT.[38,39] Despite its prognostic importance, few studies stratify by the extent of vascular involvement, primarily because MVI is currently a contraindication to LT outside of experimental settings. Choi *et al.* alluded to this in their study of living donor

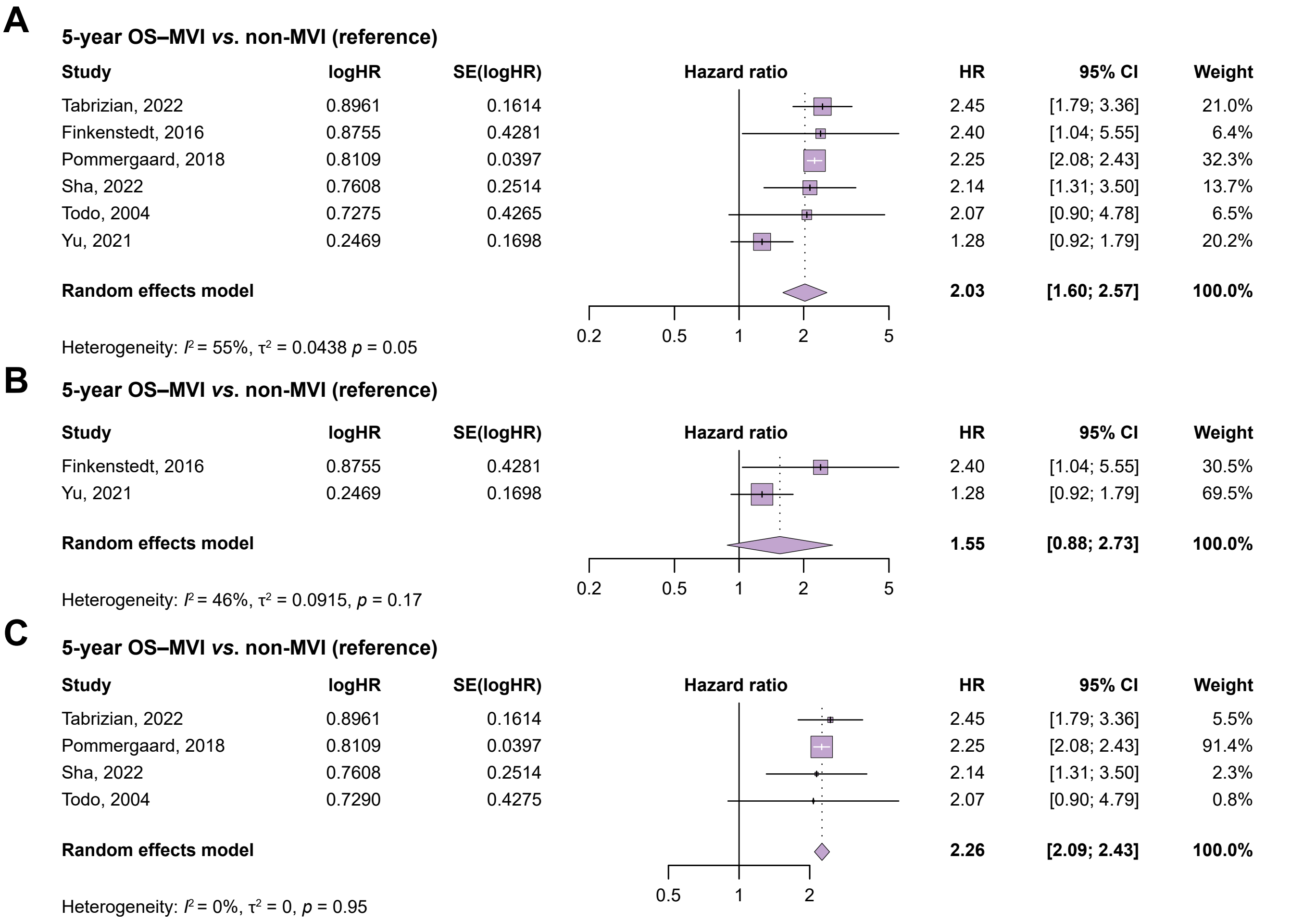

Fig. 2. Five-year OS in patients with HCC undergoing LT, stratified by presence/absence of downstaging. (A) Five-year OS for patients with HCC after LC comparing MVI *vs.* non-MVI. HRs and 95% CIs were pooled using a random-effects meta-analysis (DerSimonian and Laird method). Statistical significance: pooled HR = 2.03, 95% CI 1.60–2.57, p <0.001. Heterogeneity across studies: I^2 = 55%, τ^2 = 0.0438, p = 0.05. (B) Five-year OS for patients with HCC after LT comparing MVI having received downstaging *vs.* non-MVI. Pooled HRs were calculated using a random-effects model. Statistical significance: pooled HR = 1.55, 95% CI 0.88–2.73, p = 0.13. Heterogeneity: I^2 = 46%, τ^2 = 0.0915, p = 0.17. (C) Five-year OS of subgroup patients with HCC with no explicit mentioning of downstaging after LT comparing MVI *vs.* non-MVI. Random-effects meta-analysis was applied. Statistical significance: pooled HR = 2.26, 95% CI 2.09–2.43, p <0.001. Heterogeneity: I^2 = 0%, τ^2 = 0, p = 0.95. HCC, hepatocellular carcinoma; HR, hazard ratio; LT, liver transplant; MVI, macrovascular invasion; OS, overall survival.

LT, comparing patients without MVI to those with segmental MVI, finding no significant difference in OS or RFS.[40,41] The small sample sizes for patients receiving LT in the context of PVTT limit generalizability. Leveraging the living donor population could help corroborate these results without impacting the deceased donor waiting list.

Studies included in this meta-analysis differed significantly on pertinent and potentially confounding variables, including type and duration of locoregional or systemic treatment, extent of vascular invasion, and criteria used to assess treatment response. Protocolization of downstaging treatment and standardization of vascular assessment are essential to facilitate meaningful interinstitutional comparison. Integral to these discussions will be the multifactorial assessment of tumor biology, in which liquid biopsy might emerge as an additional diagnostic tool. Furthermore, the evolving landscape of precision medicine and immunotherapy in HCC holds promise for more effective downstaging therapy. Prospective trials registered on clinicaltrials.gov assessing systemic therapy in downstaging protocols include the ImmunoXXL (NCT05879328), iPLENTY-pvtt (NCT05339581), PLENTY202001 (NCT04425226), and ESR-20-21010 (NCT05027425) trials. Patients eligible for these trials include those with: mild to moderately differentiated disease based on core biopsy; presence of radiologic portal vein invasion (Vp1-Vp3; excluding main trunk Vp4), age 18–75 years, absence of extrahepatic spread (cN0 and cM0; thin-sliced high-resolution whole-body computed tomography); AFP <5,000 ng/ml; total tumor volume <250 cm^3; and normal hepatic synthetic function values (Child-Pugh Turcotte Score <B8). These trials will inform and potentially tailor downstaging treatment for patients with HCC and MVI.

Our findings must be viewed in the context of several limitations. First, downstaging treatment was used inconsistently within and across studies. Only two of the five studies implemented universal or near-universal pretreatment, yet details regarding treatment modalities, treatment frequency, and criteria for treatment completion were neither explicitly stated nor consistently reported across studies. Second, the number of patients with MVI undergoing LT was small, raising the possibility of selection and sample size bias. Similarly, the

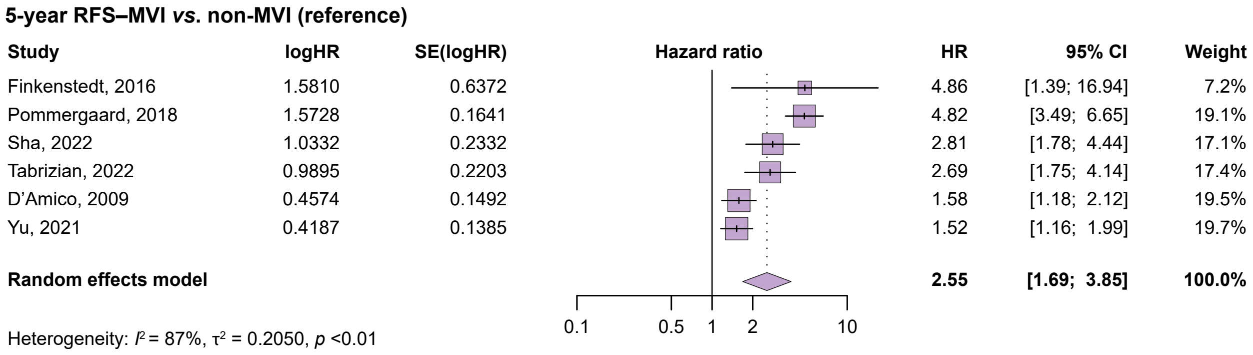

Fig. 3. Five-year RFS of patients with HCC having received LT comparing MVI *vs.* non-MVI. HRs were pooled using a random-effects meta-analysis. Statistical significance: pooled HR = 2.55, 95% CI 1.69–3.85, p <0.001. Heterogeneity was high: I^2 = 87%, τ^2 = 0.2050, p <0.01. HCC, hepatocellular carcinoma; HR, hazard ratio; LT, liver transplant; MVI, macrovascular invasion; RFS, recurrence-free survival.

number of patients with PVTT who did not undergo transplant following downstaging was only reported in one study. The dropout rate is a significant and pertinent confounding variable that could affect the observed OS and RFS and should be included in prospective trials. Third, data on crucial independent predictors, such as the level and extent of tumor thrombus, were only available in two studies. In addition, included studies varied in endpoint selection, precluding effective comparison across studies. Furthermore, many studies used surrogate endpoints, such as time to progression and progression free survival, which poorly correlate with OS. Effective treatment evaluation and knowledge translation are contingent on establishing consensus in endpoint assessment across randomized controlled trials and preferentially selecting hard endpoints for quantitative analysis.[42] Finally, included studies pre-date standard use of immune check point inhibitors, such as atezolizumab. Therefore, we were unable to comment on whether more effective downstaging is possible with these novel therapies. To enhance the robustness and generalizability of future research in this area, it is essential to address these limitations through standardized protocols, larger sample sizes, and prospective study designs.

In conclusion, the role of LT in patients with HCC with MVI is evolving. This meta-analysis provides preliminary but encouraging evidence indicating that, in carefully selected patients, effective downstaging could achieve survival outcomes approaching those without MVI. Given the heterogeneity of current data, these findings are not sufficient on their own to broadly recommend LT for patients with downstaged HCC with MVI. Further studies are essential to validate these results and to clarify which downstaging approaches and tumor characteristics are most likely to confer a transplant benefit.

Affiliations

[1]*HBP & Multi-Organ Transplant Program, University Health Network, Toronto, ON, Canada;* [2]*Department of Visceral Surgery and Medicine, Inselspital, Bern University Hospital, University of Bern, Bern, Switzerland;* [3]*Division of Gastroenterology and Hepatology, Toronto General Hospital, Toronto, ON, Canada;* [4]*Department of Hepatology, Gastroenterology, Endocrinology & Infectious Diseases, Hannover Medical School, Hannover, Germany;* [5]*Princess Margaret Cancer Center, University Health Network, Toronto, ON, Canada;* [6]*University of Toronto, Toronto, ON, Canada;* [7]*St Vincent's University Hospital and School of Medicine, University College Dublin, Dublin, Ireland*

Abbreviations

AFP, alpha-fetoprotein; aHR, adjusted hazard ratio; BCLC, Barcelona Clinic Liver Cancer; cirr, cirrhosis; DDLT, deceased donor liver transplantation; FU, follow-up; HCC, hepatocellular carcinoma; HR, hazard ratio; LDLT, living donor liver transplantation; LRT, locoregional treatment; LT, liver transplant; MC, Milan Criteria; MELD, model of end-stage liver disease; MeSH, Medical Subject Headings; mOS, median overall survival; MVI, macrovascular invasion; NASH, non-alcoholic fatty liver disease; NOS, Newcastle-Ottawa Scale; OR, odds ratio; OS, overall survival; pCR, pathologic complete response; PEI, percutaneous ethanol injection; PET, positron emission tomography; PRISMA, Preferred Reporting Items for Systematic Reviews and Meta-Analyses; PVTT, portal vein tumor thrombus; RFA, radiofrequency ablation; RFS, recurrence-free survival; SBRT, stereotactic radiotherapy; TACE, transarterial chemoembolization; TARE, transarterial radioembolization.

Financial support

The authors did not receive any financial support to produce this manuscript.

Conflicts of interest

AV has been directly paid honoraria and for consulting or advisory roles for AstraZeneca, BeiGene, Boehringer Mannheim, BMS, BTG, EISAI, GSK, Incyte, Ipsen, MSD, Hoffmann-La Roche, Servier, Sirtex, and Taiho. GMO'K has received grants from Roche and AstraZeneca; consulting fees from AstraZeneca, Servier, and Incyte; payment for lectures from Roche; and support for attending meetings from MSD and Roche. GS has received consultancy fees for AstraZeneca, Roche, Novartis, Integra, and HepaRegeniX; financial compensation for talks for Roche, AstraZeneca, Chiesi, and Integra; a grant from Roche; and has research collaborations with AstraZeneca, Natera, Roche, Stryker and HeparegeniX. The remaining authors have any conflicts of interest to disclose.

Please refer to the accompanying ICMJE disclosure forms for further details.

Authors' contributions

Study design: FL, CTJM, ME, GS. Literature review: FL, CTJM. Literature search: ME. Data extraction: FL, CTJM, FDG. Data analysis: WJC. Data interpretation: all authors. Writing: FL, CTJM, GS. Critical revision and approval: all authors.

Data availability

The datasets generated and/or analyzed during the current study are not publicly available but are available from the corresponding author on reasonable request.

Supplementary data

Supplementary data to this article can be found online at https://doi.org/10.1016/j.jhepr.2025.101566.

References

[1] Lee YH, Hsu CY, Huang YH, et al. Vascular invasion in hepatocellular carcinoma: prevalence, determinants and prognostic impact. J Clin Gastroenterol 2014;48:734–741.
[2] Llovet JM, Bustamante J, Castells A, et al. Natural history of untreated nonsurgical hepatocellular carcinoma: rationale for the design and evaluation of therapeutic trials. Hepatology 1999;29:62–67.
[3] Cerrito L, Annicchiarico BE, Iezzi R, et al. Treatment of hepatocellular carcinoma in patients with portal vein tumor thrombosis: beyond the known frontiers. World J Gastroenterol 2019;25:4360–4382.
[4] Reig M, Forner A, Rimola J, et al. BCLC strategy for prognosis prediction and treatment recommendation: the 2022 update. J Hepatol 2022;76:681–693.
[5] Sangro B, Chan SL, Kelley RK, et al. Four-year overall survival update from the phase III HIMALAYA study of tremelimumab plus durvalumab in unresectable hepatocellular carcinoma. Ann Oncol 2024;35:448–457.
[6] Espinoza M, Muquith M, Lim M, et al. Disease etiology and outcomes after atezolizumab plus bevacizumab in hepatocellular carcinoma: post-hoc analysis of IMbrave150. Gastroenterology 2023;165:286–288.
[7] Finn RS, Galle PR, Ducreux M, et al. Efficacy and safety of atezolizumab plus bevacizumab vs. sorafenib in hepatocellular carcinoma with main trunk and/or contralateral portal vein invasion in IMbrave150. Liver Cancer 2024;13:655–668.
[8] Cheng AL, Qin S, Ikeda M, et al. Updated efficacy and safety data from IMbrave150: atezolizumab plus bevacizumab vs. sorafenib for unresectable hepatocellular carcinoma. J Hepatol 2022;76:862–873.
[9] Finn RS, Qin S, Ikeda M, et al. Atezolizumab plus bevacizumab in unresectable hepatocellular carcinoma. N Engl J Med 2020;382:1894–1905.
[10] Galle PR, Decaens T, Kudo M, et al. Nivolumab (NIVO) plus ipilimumab (IPI) vs. lenvatinib (LEN) or sorafenib (SOR) as first-line treatment for unresectable hepatocellular carcinoma (uHCC): first results from CheckMate 9DW. J Clin Oncol 2024;42:LBA4008.
[11] Vogel A, Chan SL, Ren Z, et al. Camrelizumab plus rivoceranib vs. sorafenib as first-line therapy for unresectable hepatocellular carcinoma (uHCC): final overall survival analysis of the phase 3 CARES-310 study. J Clin Oncol 2024;42:4110.
[12] Llovet JM, Ducreux M, Lencioni R, et al. EASL-EORTC Clinical Practice Guidelines: management of hepatocellular carcinoma. J Hepatol 2012;56:908–943.
[13] Heimbach JK, Kulik LM, Finn RS, et al. AASLD guidelines for the treatment of hepatocellular carcinoma. Hepatology 2018;67:358–380.
[14] Vogel A, Martinelli E, Cervantes A, et al. Updated treatment recommendations for hepatocellular carcinoma (HCC) from the ESMO Clinical Practice Guidelines. Ann Oncol 2021;32:801–805.
[15] Tabrizian P, Holzner ML, Mehta N, et al. Ten-year outcomes of liver transplant and downstaging for hepatocellular carcinoma. JAMA Surg 2022;157:779–788.
[16] Liu J, Qian J, Yang Z, et al. Patients with hepatocellular carcinoma and portal vein tumour thrombosis after successful downstaging may be candidates for liver transplantation: a meta-analysis. J Hepatol 2024;80:e222.
[17] Page MJ, McKenzie JE, Bossuyt PM, et al. The PRISMA 2020 statement: an updated guideline for reporting systematic reviews. BMJ 2021;372:n71.
[18] Higgins JPT, Thomas J, Chandler J, et al., editors. Cochrane Handbook for systematic reviews of Interventions. London: Cochrane; 2019.
[19] Higgins J, Lasserson T, Thomas J, et al., editors. Methodological Expectations of Cochrane Intervention reviews. London: Cochrane; 2023.
[20] Rethlefsen ML, Page MJ. PRISMA 2020 and PRISMA-S: common questions on tracking records and the flow diagram. J Med Libr Assoc 2022;110:253–257.
[21] McGowan J, Sampson M, Salzwedel DM, et al. PRESS peer review of electronic search strategies: 2015 guideline statement. J Clin Epidemiol 2016;75:40–46.
[22] Hocking R. Yale MeSH analyzer. J Can Health Libraries Assoc 2017;38:125–126.
[23] Wells G, Shea B, O'Connell D, et al. The Newcastle-Ottawa Scale (NOS) for assessing the quality if nonrandomized studies in meta-analyses. www.ohri.ca/programs/clinical_epidemiology/oxford.asp [Accessed September 2, 2025].
[24] Higgins JPT, Thompson SG, Deeks JJ, et al. Measuring inconsistency in meta-analyses. Br Med J 2003;327:557–560.
[25] meta Schwarzer G. An R package for meta-analysis. R News 2007;7:40–47.
[26] D'Amico F, Schwartz M, Vitale A, et al. Predicting recurrence after liver transplantation in patients with hepatocellular carcinoma exceeding the up-to-seven criteria. Liver Transpl 2009;15:1278–1287.
[27] Pommergaard HC, Rostved AA, Adam R, et al. Vascular invasion and survival after liver transplantation for hepatocellular carcinoma: a study from the European Liver Transplant Registry. HPB 2018;20:768–775.
[28] Kim YT, Lee JG, Joo DJ, et al. Liver-directed combined radiotherapy for downstaging of the Milan advanced hepatocellular carcinoma converting to liver transplantation. Int J Radiat Oncol Biol Phys 2023;117:e308.
[29] Pommergaard HC, Rostved AA, Adam R, et al. Locoregional treatments before liver transplantation for hepatocellular carcinoma: a study from the European Liver Transplant Registry. Transpl Int 2018;31:531–539.
[30] Soin AS, Bhangui P, Kataria T, et al. Experience with LDLT in patients with hepatocellular carcinoma and portal vein tumor thrombosis post-downstaging. Transplantation 2020;104:2334–2345.
[31] Sha M, Chen C, Shen C, et al. Clinical analysis of deceased donor liver transplantation in the treatment of hepatocellular carcinoma with segmental portal vein tumor thrombus: a long-term real-world study. Front Oncol 2022;12:1–11.
[32] Finkenstedt A, Vikoler A, Portenkirchner M, et al. Excellent post-transplant survival in patients with intermediate stage hepatocellular carcinoma responding to neoadjuvant therapy. Liver Int 2016;36:688–695.
[33] Yu J, Zhuang L, Liu P, et al. Long-term outcomes of deceased donor liver transplantation in hepatocellular carcinoma patients with portal vein tumor thrombus: a multicenter study. Eur J Surg Oncol 2022;48:121–132.
[34] Todo S, Furukawa H. Living donor liver transplantation for adult patients with hepatocellular carcinoma. Ann Surg 2004;240:451–461.
[35] Shi J, Lai ECH, Li N, et al. A new classification for hepatocellular carcinoma with portal vein tumor thrombus. J Hepatobiliary Pancreat Sci 2011;18:74–80.
[36] Jeong Y, Shin MH, Yoon SM, et al. Liver transplantation after transarterial chemoembolization and radiotherapy for hepatocellular carcinoma with vascular invasion. J Gastrointest Surg 2017;21:275–283.
[37] Ju MR, Yopp AC. Evolving thresholds for liver transplantation in hepatocellular carcinoma: a Western experience. Ann Gastroenterol Surg 2020;4:208–215.
[38] Anon. The general rules for the clinical and pathological study of primary liver cancer. Jpn J Surg 1989;19:98–129.
[39] Kudo M. Management of hepatocellular carcinoma in Japan as a world-leading model. Liver Cancer 2018;7:134–147.
[40] Shin MH, Jeong YR, Song GW, et al. Living donor liver transplantation after combined transarterial chemoembolization and radiotherapy for hepatocellular carcinoma with major vascular invasion. HPB 2016;18:e579.
[41] Choi HJ, Kim DG, Na GH, et al. The clinical outcomes of patients with portal vein tumor thrombi after living donor liver transplantation. Liver Transpl 2017;23:1023–1031.
[42] Llovet JM, Villanueva A, Marrero JA, et al. Trial design and endpoints in hepatocellular carcinoma: AASLD Consensus Conference. Hepatology 2021;73:158–191.

Keywords: Hepatocellular carcinoma; Liver transplant; Expanded criteria; Portal vein tumor thrombus; Downstaging.
Received 6 October 2024; received in revised form 13 August 2025; accepted 19 August 2025; Available online 28 August 2025

Journal of Hepatology, Volume *8*

Supplemental information

Liver transplantation for HCC with macrovascular invasion: A systematic review and meta-analysis of observational studies

Farah Ladak, Christian Tibor Josef Magyar, Felipe D. Gaviria, Woo Jin Choi, Anudari Zorigtbaatar, Roxana Bucur, Nadia Rukavina, Arndt Vogel, Grainne Mary O'Kane, Zhihao Li, Marina Englesakis, and Gonzalo Sapisochin

Liver transplantation for HCC with macrovascular invasion: A systematic review and meta-analysis of observational studies☆

Farah Ladak, Christian Tibor Josef Magyar, Felipe D. Gaviria, Woo Jin Choi, Anudari Zorigtbaatar, Roxana Bucur, Nadia Rukavina, Arndt Vogel, Grainne Mary O'Kane, Zhihao Li, Marina Englesakis, Gonzalo Sapisochin

Table of content

Table S1 2

Table S1: Search Strategy

Search Topic: Hepatocellular Carcinoma and Liver Transplant and Macrovascular Invasion or Thrombus; limited to English language, human subjects. Conference and non-journal materials removed when possible.

Date Completed: Tuesday, January 24, 2023

MEDLINE

Ovid MEDLINE(R) 1946 to January 20, 2023

#	Searches	Results
1	Carcinoma, Hepatocellular/	101701
2	Adenoma, Liver Cell/	1015
3	Liver Neoplasms, Experimental/	15329
4	exp Liver Neoplasms/ and (adenoma* or carcinom* or hepatoma*).mp.	127106
5	(adult? adj1 liver? adj1 cancer*).mp.	10
6	(cancer* adj3 (liver* or hepat*)).mp,kf.	39958
7	(carcinom* adj3 (liver* or hepat*)).mp,kf.	127015
8	experimental hepatoma?.mp.	87
9	experimental hepat* neoplasm?.mp.	1
10	experimental liver neoplasm?.mp.	2
11	((hepatocellular or liver?) adj3 adenoma?).mp.	2533
12	(hepatocellular* adj2 carcinom*).mp.	122068
13	(liver? adj1 cell* adj1 adenoma*).mp.	1197
14	(liver? adj1 cell?? adj1 carcinom*).mp.	354
15	hepatocarcino*.mp,kw.	13026
16	hepato-carcino*.mp,kw.	128
17	HCC-MVI.mp.	13
18	morris?? hepatoma?.mp.	853
19	novikoff?? hepatoma?.mp.	483
20	or/1-19 [Hepatocellular Carcinoma & related terms]	174692
21	Liver Transplantation/	63393
22	exp Liver/tr [Use of "transplantation" floating subheading]	5
23	exp Liver Diseases/tr	6
24	exp Liver Diseases/ and exp Transplants/	1387
25	exp Liver/ and exp donor selection/	150
26	exp Liver/ and exp Graft Rejection/	1751
27	exp Liver/ and exp "Graft vs Host Disease"/	488
28	exp Liver/ and Tissue Donors/	1320
29	exp Liver/ and exp "Tissue and Organ Harvesting"/	577
30	exp Liver/ and exp "Tissue and Organ Procurement"/	578
31	exp Liver/ and exp Transplants/	745

32	exp Liver/ and exp Transplant Donor Site/	10
33	exp Liver/ and exp Unrelated Donors/	5
34	exp Liver/ and exp Vascular Grafting/	1638
35	exp Liver Diseases/ and exp Donor Selection/	558
36	exp Liver Diseases/ and exp Graft Rejection/	2918
37	exp Liver Diseases/ and exp "Graft vs Host Disease"/	694
38	exp Liver Diseases/ and exp "Tissue and Organ Harvesting"/	881
39	exp Liver Diseases/ and exp "Tissue and Organ Procurement"/	1750
40	exp Liver Diseases/ and exp Transplant Donor Site/	9
41	exp Liver Diseases/ and exp Unrelated Donors/	10
42	exp Liver Diseases/ and exp Vascular Grafting/	7960
43	Tissue Donors/ and (liver? or hepat*).mp.	7027
44	tr.fs. and (liver? or hepat*).mp. [Use of "transplantation" floating subheading]	6440
45	(deceased adj2 donor*).mp.	6925
46	(liver? adj3 transplant*).mp.	78878
47	(liver? adj3 allograft*).mp.	2898
48	(liver? adj3 autograft*).mp.	18
49	(liver? adj3 autotransplan*).mp.	132
50	(liver? adj3 auto-transplan*).mp.	6
51	(liver? adj3 graft*).mp.	6603
52	(living adj3 donor?).mp.	23409
53	(hepat* adj3 transplant*).mp.	10566
54	(hepat* adj3 allograft*).mp.	745
55	(hepat* adj3 autograft*).mp.	9
56	(hepat* adj3 autotransplan*).mp.	44
57	(hepat* adj3 auto-transplan*).mp.	4
58	(hepat* adj3 graft*).mp.	1317
59	(live adj3 donor?).mp.	2493
60	(liver? adj3 donor?).mp.	10886
61	(liver? adj3 donat*).mp.	863
62	(hepat* adj3 donor?).mp.	2389
63	(hepat* adj3 donat*).mp.	137
64	hemiliver?.mp.	165
65	hemihepatectom*.mp.	981
66	or/21-65 [Liver Transplantation & related terms]	117309
**67**	**20 and 66 [HCC + Liver Transplant]**	**12513**
68	exp Thrombosis/ and exp Portal Vein/	4755
69	exp Venous Thrombosis/	59323
70	thromb*.mp.	518841
71	HCC-MVI.mp.	13

72	PVTT.mp.	507
73	(arterial* adj3 inva*).mp.	1513
74	((artery or arteries) adj3 inva*).mp.	1999
75	(vasc* adj3 inva*).mp.	11133
76	(macrovasc* adj3 inva*).mp.	340
77	(venous adj3 inva*).mp.	2544
78	(macroscop* adj3 inva*).mp.	421
79	(blood adj2 clot*).mp.	10811
80	(major adj3 inva*).mp.	1383
81	or/68-80 [Thrombosis or Macrovascular Invasion]	549394
**82**	**67 and 81 [HCC + Liver Transplant + Thrombosis or Macrovascular Invasion]**	**1152**
	Limits applied:	
83	limit 82 to english language	1035
84	83 not (exp animals/ not (exp animals/ and exp humans/))	1027
85	limit 83 to humans	1027
86	84 or 85	1027
87	86 not (animal or animals or ape or apes or baboon or baboons or bat or bats or beagle or beagles or bird or birds or boar or boars or bonobo or bonobos or bovine or camel or camels or canine or canines or cat or cats or cattle or chick or chicks or chicken or chickens or chimpanzee or chimpanzees or dog or dogs or dromedary or dromedaries or duck or ducks or equine or equines or feline or felines or ferret or ferrets or frog or frogs or fowl or fowls or goat or goats or hamster or hamsters or hare or hares or hen or hens or horse or horses or lamb or lambs or livestock or macaque or macaques or mandrill or mandrills or mice or mink or minks or monkey or monkeys or mouse or murine or ovine or pig or pigs or piglet or piglets or poultry or porcine or orangutan or orangutans or rabbit or rabbits or rat or rats or rodent or rodents or sheep or spaniel or spaniels or swine or tamarin or tamarins or tiger or tigers or veterinary or veterinarian or veterinarians or waterfowl or waterfowls or weasel or weasels or veterinar* or fish or shellfish).ti,jw.	1023
88	86 not 87 [double check]	4
**89**	**87**<br>**[HCC + Liver Transplant + Thrombosis or Macrovascular Invasion; limited to English, humans]**	**1023**

Embase

Embase Classic+Embase 1947 to 2023 January 20

#	Searches	Results
1	exp liver cell carcinoma/ [Used for HCC in Embase]	198029
2	Carcinoma, Hepatocellular/ [MeSH]	112715
3	Adenoma, Liver Cell/	3301
4	exp liver cancer/ and (adenom* or carcinom* or hepatoma*).mp.	230402
5	exp Liver Neoplasms/ and (adenoma* or carcinom* or hepatoma*).mp.	240153
6	(adult? adj1 liver? adj1 cancer*).mp.	14

7	(cancer* adj3 (liver* or hepat*)).mp,kw.	95613
8	(carcinom* adj3 (liver* or hepat*)).mp,kw.	234254
9	experimental hepatoma?.mp.	116
10	experimental hepat* neoplasm?.mp.	1
11	experimental liver neoplasm?.mp.	328
12	((hepatocellular or liver?) adj3 adenoma*).mp.	5527
13	(hepatocellular* adj2 carcinom*).mp.	168492
14	hepatocarcino*.mp,kw.	19239
15	hepato-carcino*.mp,kw.	237
16	HCC-MVI.mp.	27
17	(liver? adj1 cell?? adj1 adenoma*).mp.	382
18	(liver? adj1 cell?? adj1 carcinom*).mp.	197983
19	morris?? hepatoma?.mp.	1197
20	novikoff?? hepatoma?.mp.	886
21	or/1-20 [Hepatocellular Carcinoma & related terms]	324113
22	Liver Transplantation/	116268
23	exp Liver Diseases/ and exp Transplants/	125050
24	exp Liver/ and exp donor selection/	208
25	exp Liver/ and exp Graft Rejection/	3759
26	exp Liver/ and exp "Graft vs Host Disease"/	2517
27	exp Liver/ and Tissue Donors/	7754
28	exp Liver/ and exp "Tissue and Organ Harvesting"/	14
29	exp Liver/ and exp "Tissue and Organ Procurement"/	46144
30	exp Liver/ and exp Transplants/	46144
31	exp Liver/ and exp Transplant Donor Site/	2188
32	exp Liver/ and exp Unrelated Donors/	96
33	exp Liver/ and exp Vascular Grafting/	792
34	exp Liver Diseases/ and exp Donor Selection/	938
35	exp Liver Diseases/ and exp Graft Rejection/	16283
36	exp Liver Diseases/ and exp "Graft vs Host Disease"/	6649
37	exp Liver Diseases/ and exp "Tissue and Organ Harvesting"/	15
38	exp Liver Diseases/ and exp "Tissue and Organ Procurement"/	125050
39	exp Liver Diseases/ and exp Transplant Donor Site/	5505
40	exp Liver Diseases/ and exp Unrelated Donors/	349
41	exp Liver Diseases/ and exp Vascular Grafting/	2126
42	Living Donors/ and (liver? or hepat*).mp.	14311
43	exp Tissue Donors/ and (liver? or hepat*).mp.	51644
44	(deceased adj2 donor*).mp.	20482
45	(liver? adj3 transplant*).mp.	152729

46	(liver? adj3 allograft*).mp.	4841
47	(liver? adj3 autograft*).mp.	28
48	(liver? adj3 autotransplan*).mp.	205
49	(liver? adj3 auto-transplan*).mp.	26
50	(liver? adj3 graft*).mp.	43775
51	(hepat* adj3 transplant*).mp.	18556
52	(hepat* adj3 allograft*).mp.	1015
53	(hepat* adj3 autograft*).mp.	18
54	(hepat* adj3 autotransplan*).mp.	74
55	(hepat* adj3 auto-transplan*).mp.	6
56	(hepat* adj3 graft*).mp.	2338
57	(live adj3 donor?).mp.	5927
58	(liver? adj3 donor?).mp.	22713
59	(liver? adj3 donat*).mp.	1901
60	(living adj3 donor?).mp.	43893
61	(hepat* adj3 donor?).mp.	4707
62	(hepat* adj3 donat*).mp.	261
63	hemiliver?.mp.	345
64	hemihepatectom*.mp.	2871
65	hemi*hepatectom*.mp.	2871
66	liver graft/ [Embase]	33330
67	exp deceased donor/	9186
68	exp liver/ and exp transplantation/ [Embase]	46144
69	exp liver/ and organ transplantation/	2545
70	exp liver disease/ and exp transplantation/	125050
71	exp liver disease/ and organ transplantation/	4318
72	exp liver resection/	17152
73	exp living donor/ and (liver? or hepat*).mp.	17510
74	or/22-73 [Liver Transplantation & related terms]	280285
**75**	**21 and 74 [HCC + Liver Transplant]**	**39680**
76	exp vein thrombosis/	162742
77	blood clot/	8255
78	deep vein thrombosis/	74163
79	lung embolism/	120708
80	thrombi.mp.	25421
81	thrombos*.mp.	449977
82	thrombot*.mp.	87129
83	((pulmonary or lung?) adj2 emboli*).mp.	133799

84	((artery or arteries) adj3 inva*).mp.	3474
85	(arterial* adj3 inva*).mp.	2916
86	(macro* adj3 inva*).mp.	4602
87	(major* adj3 inva*).mp.	3100
88	(vascular* adj3 inva*).mp.	20450
89	(portal vein? adj2 inva*).mp.	1544
90	PVTT.mp.	1091
91	HCC-MVI.mp.	27
92	(venous adj3 inva*).mp.	4314
93	(blood adj2 clot?).mp.	62378
94	exp thrombosis/	456994
95	or/76-94 [Thrombosis / Invasion]	717814
**96**	**75 and 95 [HCC + Liver Transplant + Vascular Invasion]**	**4353**
	Limits applied:	
97	limit 96 to english language	4184
98	97 not ((exp animals/ or exp animal experimentation/ or nonhuman/) not ((exp animals/ or exp animal experimentation/ or nonhuman/) and exp human/))	4149
99	limit 97 to human	3928
100	98 or 99	4149
101	100 [HCC + Liver Transplant + Vascular Invasion; limited to English, human]	4149
102	remove duplicates from 101 [removal of internal duplicate citations]	4057
103	limit 102 to (conference abstracts or "preprints (unpublished, non-peer reviewed)" or (books or chapter or conference abstract or conference paper or "conference review" or "preprint (unpublished, non-peer reviewed)") or (book or book series or conference proceeding or "preprint archive (unpublished, non-peer reviewed)" or trade journal))	1629
104	102 not 103 [removal of conference and non-journal material]	2428
**105**	**104 [HCC + Liver Transplant + Vascular Invasion; limited to English, human, journal articles]**	**2428**
